# Investigating the hard X-ray production via proton spallation on different materials to detect elements

**DOI:** 10.1371/journal.pone.0288287

**Published:** 2023-08-18

**Authors:** Saeedeh Khezripour, Mohammadreza Rezaie, Mehdi Hassanpour, Marzieh Hassanpour, Mohammad Rashed Iqbal Faruque, Mayeen Uddin Khandaker

**Affiliations:** 1 Department of Molecular and Atomic Physics, Faculty of Modern Science and Technology, Graduate University of Advanced Technology, Kerman, Iran; 2 Department of Nuclear Engineering, Faculty of Modern Sciences and Technologies, Graduate University of Advanced Technology, Kerman, Iran; 3 Space Science Centre (ANGKASA), Institute of Climate Change (IPI), Universiti Kebangsaan Malaysia, Malaysia, Malaysia; 4 Centre for Applied Physics and Radiation Technologies, School of Engineering and Technology, Sunway University, Selangor, Malaysia; 5 Department of General Educational Development, Faculty of Science and Information Technology, Daffodil International University, Dhaka, Bangladesh; Mohanlal Sukhadia University, INDIA

## Abstract

Various atomic and nuclear methods use hard (high-energy) X-rays to detect elements. The current study aims to investigate the hard X-ray production rate via high-energy proton beam irradiation of various materials. For which, appropriate conditions for producing X-rays were established. The MCNPX code, based on the Monte Carlo method, was used for simulation. Protons with energies up to 1650 MeV were irradiated on various materials such as carbon, lithium, lead, nickel, salt, and soil, where the resulting X-ray spectra were extracted. The production of X-rays in lead was observed to increase 16 times, with the gain reaching 0.18 as the proton energy increases from 100 MeV to 1650 MeV. Comparatively, salt is a good candidate among the lightweight elements to produce X-rays at a low proton energy of 30 MeV with a production gain of 0.03. Therefore, it is suggested to irradiate the NaCl target with 30 MeV proton to produce X-rays in the 0–2 MeV range.

## Introduction

X-rays and their medical applications are significant in element detection [1, 2]. The X-ray scattering and diffraction (XRD) technique in crystallography can be used to analyse a crystal structure [3]. While the X-ray fluorescence (XRF) method, a non-destructive and fast technique, is widely used to determine the elemental composition of a material [4, 5]. Elements can be identified using proton or ion radiation on the target [6]. For instance, in the Proton Induced X-ray Emission (PIXE) and Proton Induced Gamma-ray Emission (PIGE) methods, the gamma and X-rays emitted from the activated materials at the nuclear and atomic levels were used for elemental analyses of the targets [7]. The generated gamma and X-rays or an ion beam are the primary tools for detecting elements. Generally, gamma and hard or soft X-rays are produced by irradiating high-energy electrons and protons on tungsten and other materials [8–12]. The terms "hard X-ray" and "gamma ray" are commonly used to distinguish between sources of electromagnetic radiation. Specifically, X-rays are emitted due to the de-excitation of atomic electron levels around nuclei.

Contrarily, de-exciting nuclear levels of protons or neutrons in nuclides produces gamma rays. There are four possible ways for photon emission to occur when protons travel through a target material, namely: (a) through the de-excitation of atomic levels (i.e., X-ray emission); (b) through the de-excitation of nuclear levels (i.e., gamma-ray emission); (c) by the stopping of protons in the target (i.e., X-ray emission); and (d) through spallation processes, whereby secondary particles produced by the proton target interaction emit photons (i.e., both X-ray and gamma-ray emission). In the present study, all four mechanisms were observed following proton irradiation of the targets. Hence, it is impossible to differentiate between X-ray and gamma-ray emission in a mixed-field environment consisting of both types of radiation. Consequently, the emitted photons resulting from spallation processes were collectively referred to as "hard X-rays" or "gamma rays," with the former term being used in this study.

The energy of generated X-rays depends on the energy of the incident electron and proton beam. For instance, electron beams of up to 150 keV energy are used in industrial X-ray devices to produce X-rays in the same range [13]. Meanwhile, the electrons beam up to 15 MeV energy are used in the Varian accelerator to produce an X-ray in the same energy range [14]. Moreover, there are many reports on using radioactive elements as sources in element detection devices [15]. The radioactive elements are primarily used as the source due to eliminating high voltage and X-ray tubes to produce portable element detection devices. The proton source is the central part of elements detection in the PIXE method, where the intensity of the X-ray emitted from the target depends on the energy of the protons [16, 17]. Also, X-rays from the proton irradiation omitted on materials can be used as an X-ray source because the intensity depends on the energy and intensity of the irradiated protons. In a previous study, a neutron beam was employed to irradiate 162Er to produce a 163Ho isotope. The sample was irradiated to activate neutron for 104 s at the PSI spallation neutron source (SINQ) facility, following which the gamma spectra of the 162Er sample containing 163Ho isotope were recorded at various intervals of 5 h, 24 h, and 4 d post-irradiation [18]. The detection of elements was achieved through gamma spectra resulting from the spallation of a target under neutron irradiation. The neutron beam production was accomplished through proton beam irradiation of the spallation target [19]. In another study, Chiera et al. [20] measured the gamma rays produced by proton and neutron spallation of various targets for element detection purposes.

The present work analyses the hard X-ray spectrum emitted from the interaction of high-energy protons with several materials, such as soil, salt, and the elements available on the earth’s surface, using the Monte Carlo method as a hard X-ray source. The hard X-ray source with proton collision can be introduced in two proton energy ranges: (a) hard X-ray source with the collision of the high-energy proton (up to 1 GeV) and (b) hard X-ray source with the collision of protons with energies less than 100 MeV. The collision of protons with different targets excites their nucleus, where the excited nucleus emits gamma rays with a specific energy spectrum [8]. Since proton absorption is possible by the target nucleus, the initial nucleus can be converted to a compound nucleus. The compound nucleus decays through various processes, including neutron emission, positive and negative beta particles, alpha particles, gamma emission, fission, etc. [21]. While increasing the proton energy may result in a spallation process for the compound nucleus [22, 23]. During the spallation process, the nucleus is converted into different elements. The spallation product is mainly at the excitation level and can be de-excited by emitting gamma and hard X-rays [24–29]. Usually, spallation has a specific threshold energy for every target [30]. The threshold energy of the spallation can be increased by increasing the element mass number. Recent studies calculated some elements’ photon production cross-section using the proton spallation process [16, 17]. The photon production rate due to the proton spallation process for many elements was also studied [31]. Hence, to choose the proper hard X-ray source in the present study based on the threshold energy for the proton spallation process of different targets, the hard X-ray energy spectrum emitted from many materials was studied using the Monte Carlo N-Particle Transport (MCNPX) code.

The MCNPX code is one of the most robust codes for nuclear calculations based on the Monte Carlo method. Several studies have used this code [32, 33] and reported experimental results versus simulations with greater accuracy [34]. This code is widely used in various fields like detectors [35], archaeology [36], food and dairy irradiation [37, 38], radiation shielding [39], and radiography and radiotherapy [40, 41]. The energy threshold and proper energy range to produce hard X-rays from proton spallation of NaCl, soil, and the well-known materials available in nature for the Photon Induced X-ray Florescence Emission (PIXFE) method [42–44] are described in this study.

## Methods

A proton beam with surface energy distribution and different energies was irradiated on a cylinder with variable height and radius to produce X-rays. The proposed geometry was imported into the MCNPX code as an input file. The FT8 command was used in this code to activate the spallation process. This code together with the RES command [45] can trigger the production process of X-rays due to proton irradiation on various targets followed by the calculation of the produced particle spectrum. To determine the spallation products in the range of atomic numbers Z_1_ to Z_2_, the command RES Z_1_ Z_2_ was given after FT8. The general command was written as follows:

F8:h

FT8 RES Z_1_ Z_2_

where the h parameter is related to the proton spallation process, Tally F8 is a type of output command that indicates the pulse height or probability of the desired quantity, and FT8 is a command that activates the secondary particles produced by the irradiation of the primary particle on the target. If the command “RES Z_1_ Z_2_” is used, the secondary particles produced in the range of atomic numbers Z_1_ to Z_2_ will be displayed in the output. The results of proton spallation products were recorded in the output file as “ZAID S R (Atomic number×1000+Math number)” by writing the above command in the input file of the MCNPX code and running it. The results reflect the characteristics of the atomic number and the mass number of spallation products, where “S” is the gain of products and “R” is the error of Monte Carlo calculations. When the number of particles for simulation was set to be 10^7^, the resulting calculation error was recorded below 1%. It is possible to calculate the hard X-ray production with high accuracy due to the spallation process. After investigating the probability of the proton spallation process, the following command was used,

F2:P

e_2_ E_min_ ni E_max_

Where “p” indicates the produced photons, “e_2_” is used to determine the spectrum of produced photons, and “ni” is the number of energy intervals. Whereas “E_min_” is zero, “E_max_” reflects the proton energy, and “ni” is the amount of each energy interval equivalent to 1 keV.

**Fig 1:**
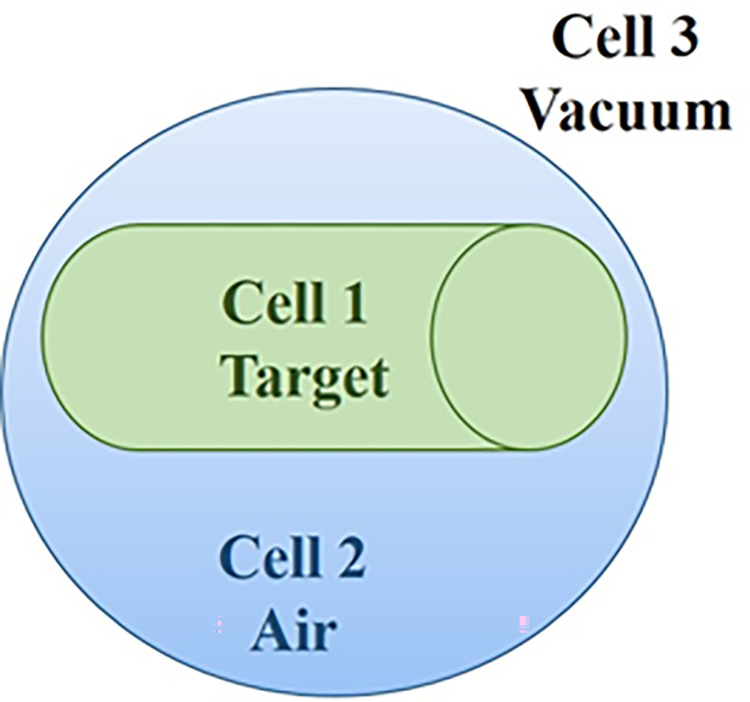
Representation of the cells defined in the MNCPX code for calculating X-ray spectrum.

Due to the limited particles’ energy level in the MCNPX code and to raise the energy of trackable particles, the PHYS command was used by defining a spherical surface around the target on which the proton was irradiated and the energy spectrum of X-ray was investigated using the *F2 command. Table 1 lists the data relating to the mass number, density, and threshold energy of materials used in this research, including soil, salt, lead, lithium, etc. While Fig 1 depicts the geometry used in the present study. Cells 1 and 2 indicate calculating the X-ray spectrum produced in the target.

**Table 1:** The consistency between simulation and experimental results of photon production due to the spallation process of light nuclei [4, 8–12, 22, 23, 54, 55].

Spallation targets	Proton energy, Ep (MeV)	Produced photon energy (MeV)		
		Simulation	Experiment	Consistency (%)
^28^Si	113	1.78	1.78	100
^19^F	113	6.14	6.13	99
^16^O	113	6.13	6.13	100
^12^C	85	0.74	0.72	97%
		1	1.02	98%
		1.41	1.38	98%
		1.67	1.64	98%
		1.88	1.88	100%
		1.95	2.00	97%
		4.45	4.44	99%
		5.25	5.27	99%
^7^Li	0.65–12	0.49	0.48	98%
Median of consistency				98%

Cell 1 is characterised as a cylinder with a variable height and material with a radius of 20 cm. The proton source is situated as a surface source within this call with a 1 cm radius on the surface of the cylinder base. Since the rays stemming from the source are emitted perpendicularly to the cylinder base, the distribution of the source’s surface is presumed to be uniform. Cell 2 which encircles the target, is classified as an air cell. Meanwhile, Cell 3 is a vacuum that envelops the outer space of the sphere measuring 40 cm in radius. In this cell, particle tracking is irrelevant and must not be considered in the simulation. The energy spectrum of the hard X-rays generated in the target reaching the surface of the cylinder’s surrounding sphere can be ascertained using the command codes *F1 or *F2.

## Results

### Validation of a Monte Carlo program for hard X-ray production by spallation

The characteristic of hard X-ray spectrum obtained from the bombardment of ^12^C by 85 MeV proton simulation result is reported and compared with the experimental results [46–48] as depicted in Table 1. The consistency between the present and experimental results was calculated for each hard X-ray peak, where the consistency was approximately 98%. Gamma-ray characteristics at 1.78 MeV energy were observed experimentally when ^28^Si nuclei were bombarded with 113 MeV protons [49, 50]. The consistency between the simulation and experimental results was estimated at 100% (Table 1). The characteristics of gamma at 6.13 MeV energy each were experimentally measured by bombarding ^19^F and ^16^O nuclei with 113 MeV protons [51, 52]. The simulation results estimated the values of 6.13 MeV and 6.14 MeV for ^16^O and ^19^F, respectively. According to Table 1, the consistency was 100% and 99% for ^16^O and ^19^F, respectively. A characteristic gamma with an energy of 0.48 MeV was measured experimentally by bombarding ^7^Li nuclei with protons in the range of 0.65 to 12 MeV energy [53]. The simulation results yielded an energy of 0.49 MeV for this range. Table 1 displays the overall consistency between the experimental and simulation results at 98%.

According to Table 1, the average consistency between simulation and experimental results was 98%. Furthermore, the collision of carbon with a proton with 3–5 MeV energy can excite it to produce gamma-rays with an energy of 4.44 MeV [56], which is 100% consistent with the simulation results.

### Hard X-ray production by proton beam spallation

**Fig 2:**
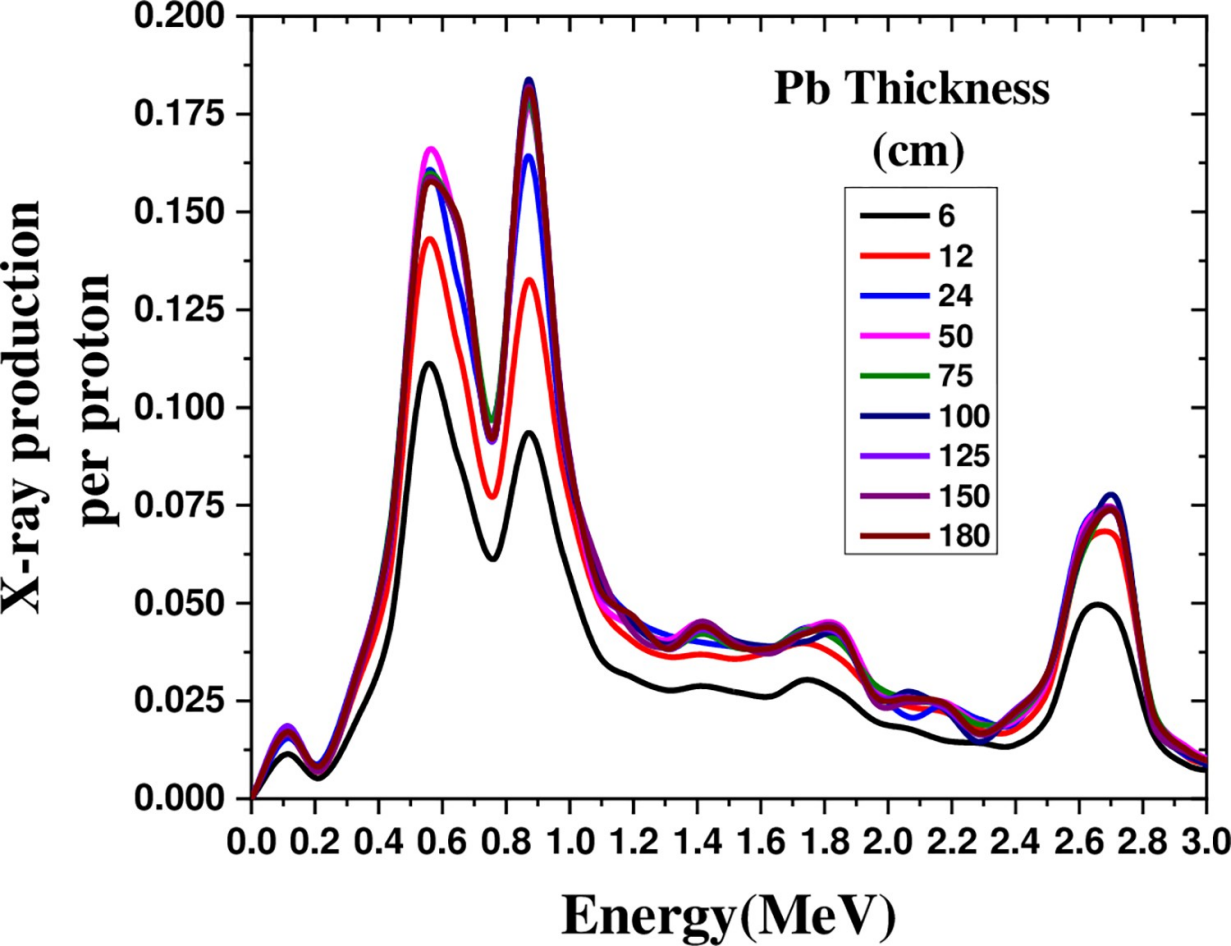
X-ray production in the energy range of 0–4 MeV by a 600 MeV proton beam irradiated on a lead cylinder with a radius of 20 cm and different cylinder heights.

The Monte Carlo method can be confidently used to check hard X-ray production with the bombardment of different nuclei by the proton beam due to its high percentage of consistency. Fig 2 illustrates the production of X-rays in the energy range of 0–4 MeV by irradiating a 600 MeV proton beam on a lead cylinder with a radius of 20 cm as a function of height (6, 12, 24, 50, 75, 100, 125, 150, and 180 cm). The X-ray production rate reached its optimal state by changing the height of the cylinder from 6 cm to 50 cm, producing three peaks at 0.54455, 0.87129, and 2.6139 MeV.

**Fig 3:**
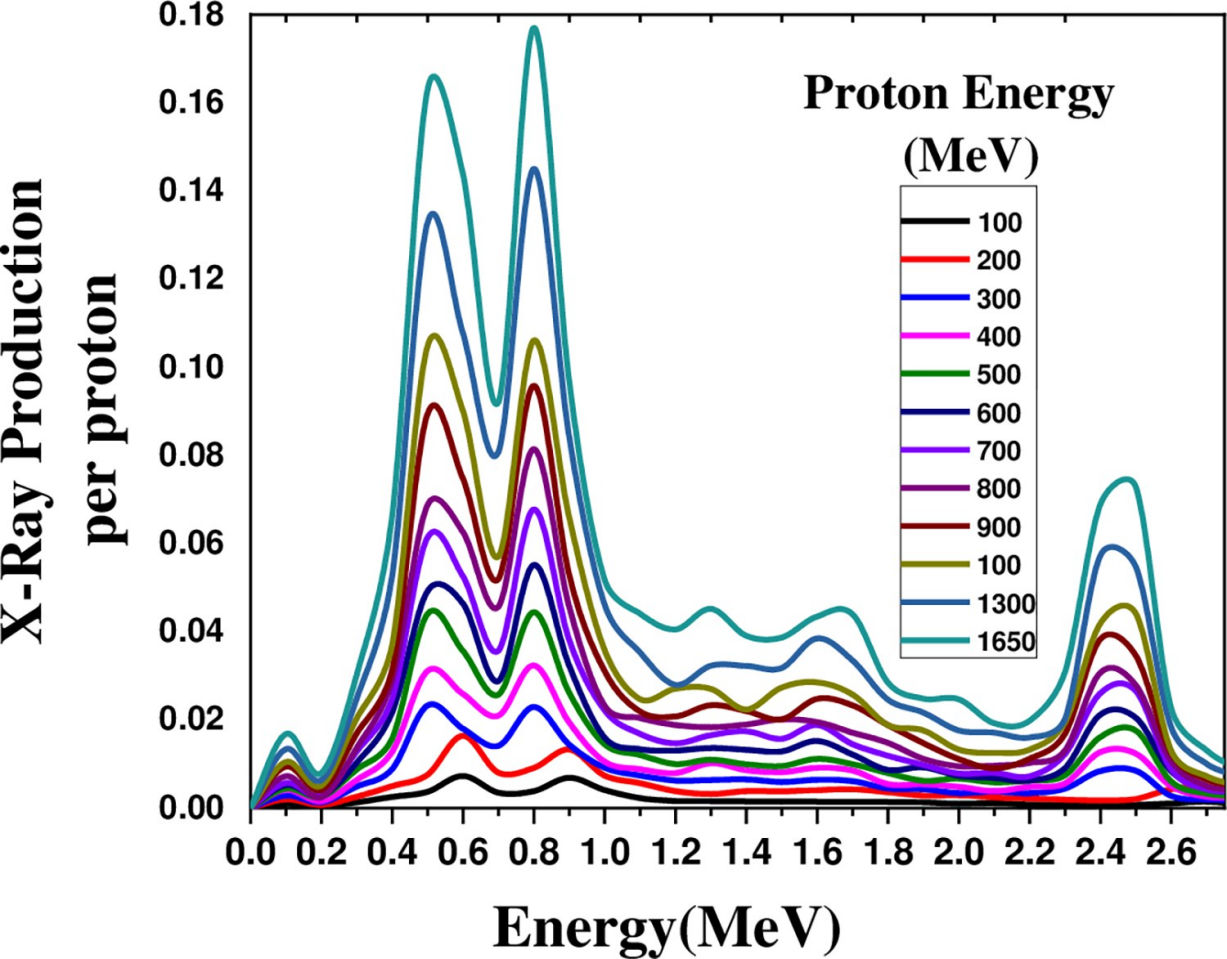
The X-ray production in the energy range of 0–4 MeV by a proton beam energy ranging from 100 to 1650 MeV, irradiated on a 50 cm lead cylinder.

Fig 3 illustrates the X-ray production in the range of 0–4 MeV by a proton beam irradiated on a lead cylinder with a height of 50 cm as a function of energy ranging from 100 to 1650 MeV. The X-ray production increased by 16 times for the 1650 MeV proton beam compared to the 100 MeV proton.

**Fig 4:**
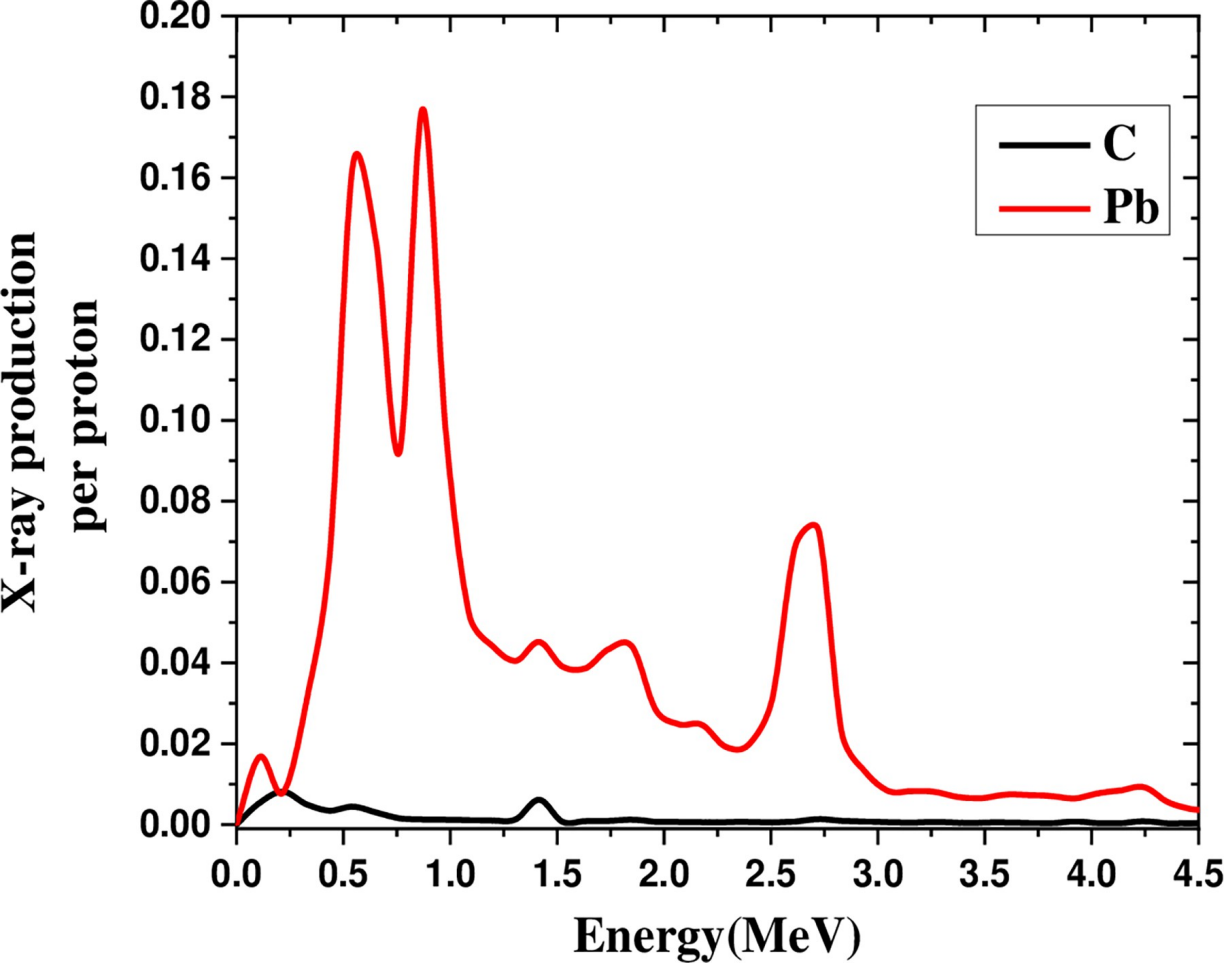
Comparison of X-ray production in the 0–6 MeV range by irradiating 600 MeV proton beam on lead and carbon.

Fig 4 depicts the X-ray production in the energy range of 0–6 MeV by irradiating a 600 MeV proton beam on lead and carbon, which produced X-rays in the lead, 16 times higher than in carbon.

**Fig 5:**
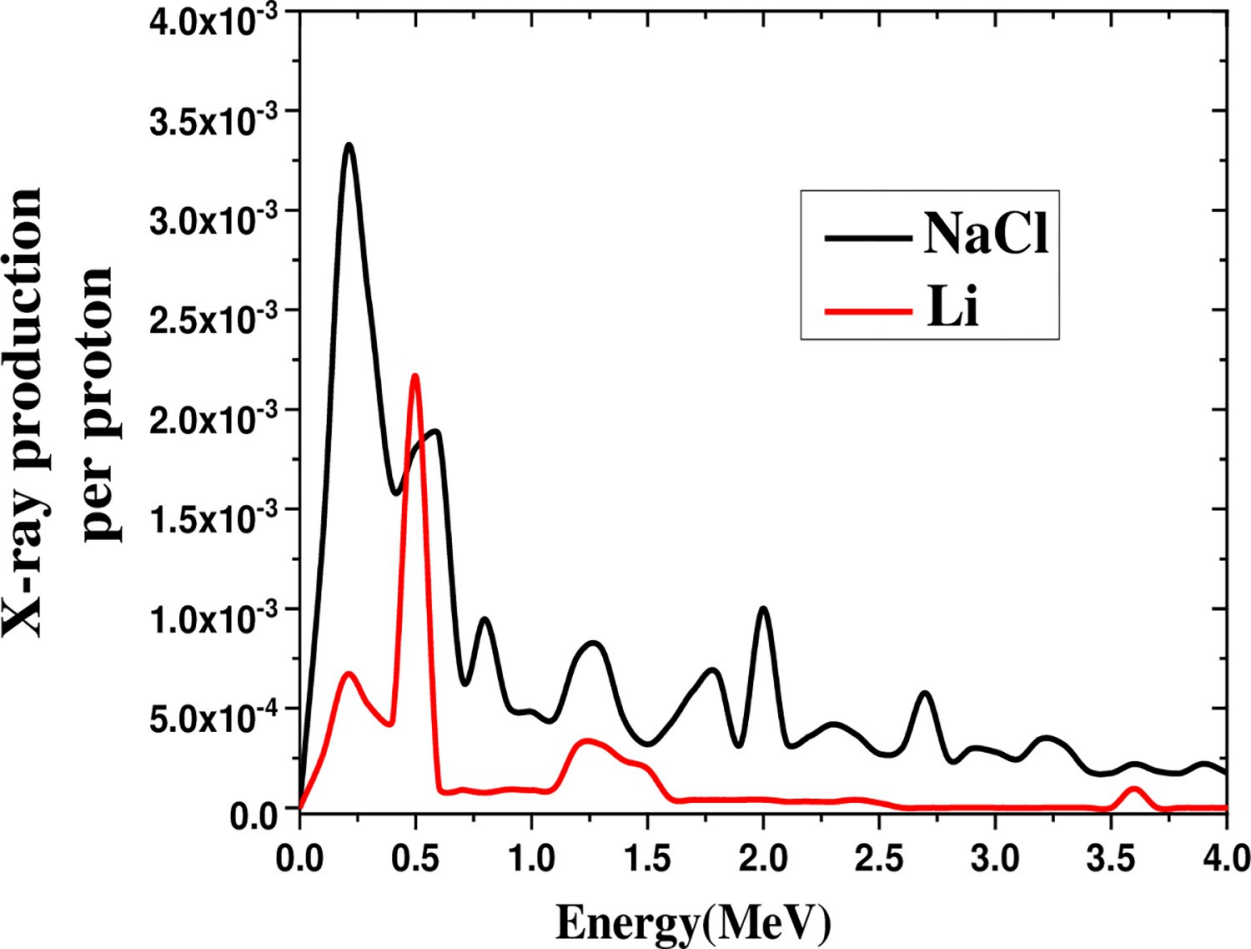
Comparison of X-ray production in the 0–8 MeV range by irradiating 40 MeV proton beam on salt and lithium.

Fig 5 represents the X-ray production in the 0–8 MeV range by a 40 MeV proton beam on salt and lithium. The salt spectrum, which peaked at 0.22 MeV, was observed to be higher than the lithium peak at 0.5 MeV.

**Fig 6:**
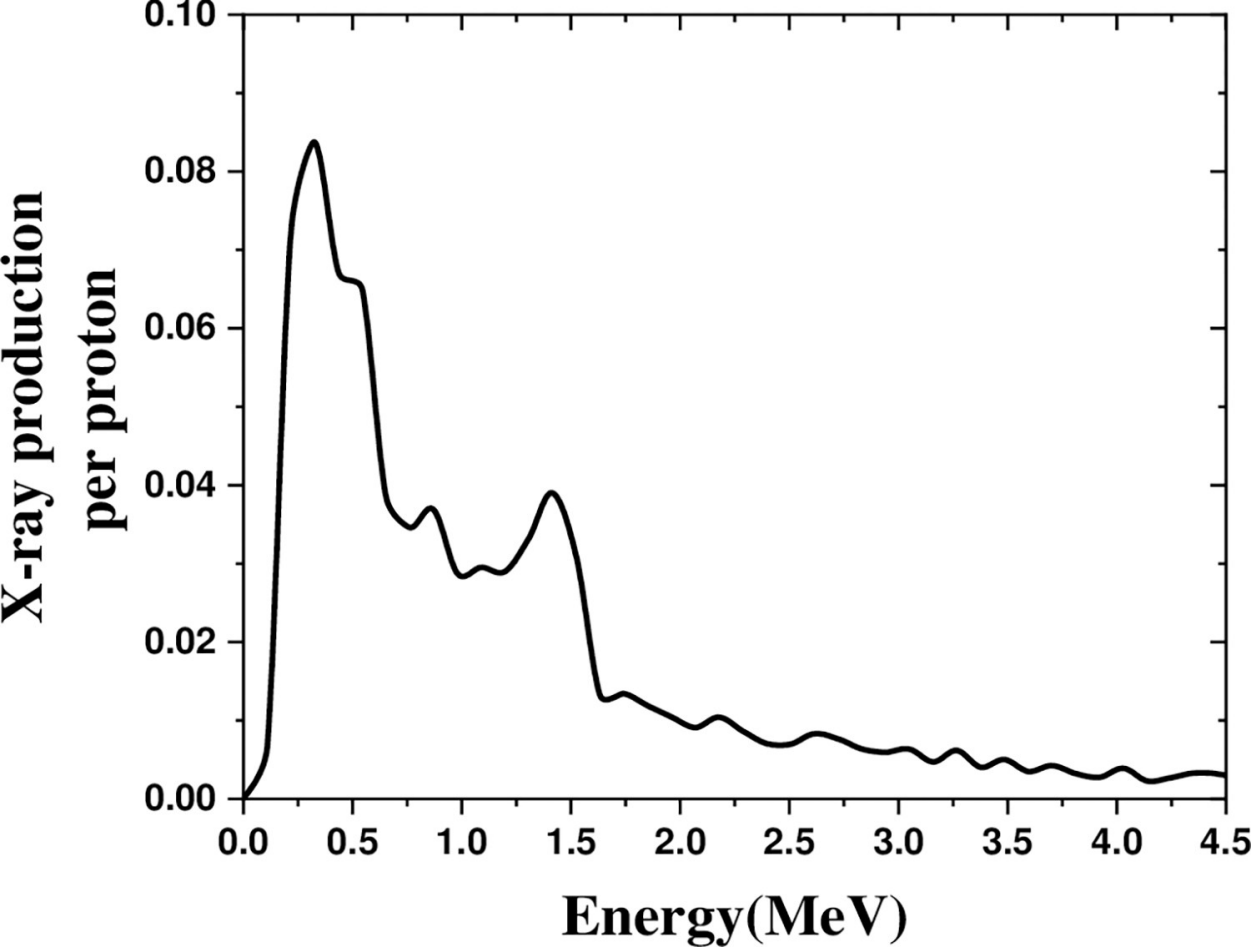
The X-ray production in the range of 0–5 MeV by irradiating 600 MeV proton beam on nickel.

Fig 6 represents the X-ray production in the energy range of 0–5 MeV by a 600 MeV proton beam on nickel. Nickel’s spectrum peaked at 0.33 MeV, with a proper X-ray production in the 0–2 MeV range.

**Fig 7:**
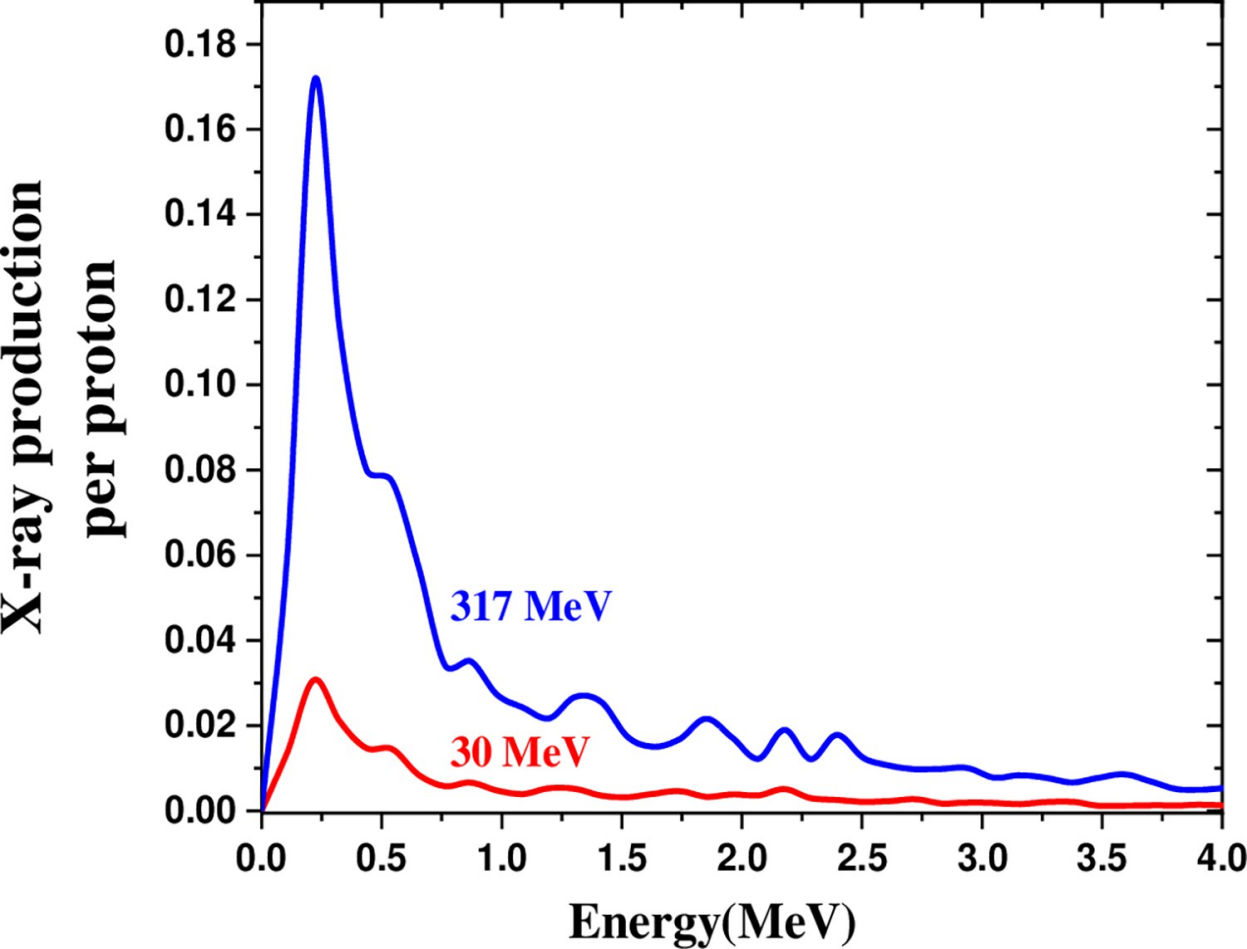
The X-ray production in the energy range of 0–5 MeV by irradiating 30 MeV and 317 MeV proton beams on salt.

Fig 7 depicts the X-ray production in the energy range of 0–5 MeV by irradiating 30 MeV and 317 MeV proton beams on salt. By increasing the proton energy by 10.57 times, the salt spectrum peaked at 0.22 MeV, nearly crossed by six times.

**Fig 8:**
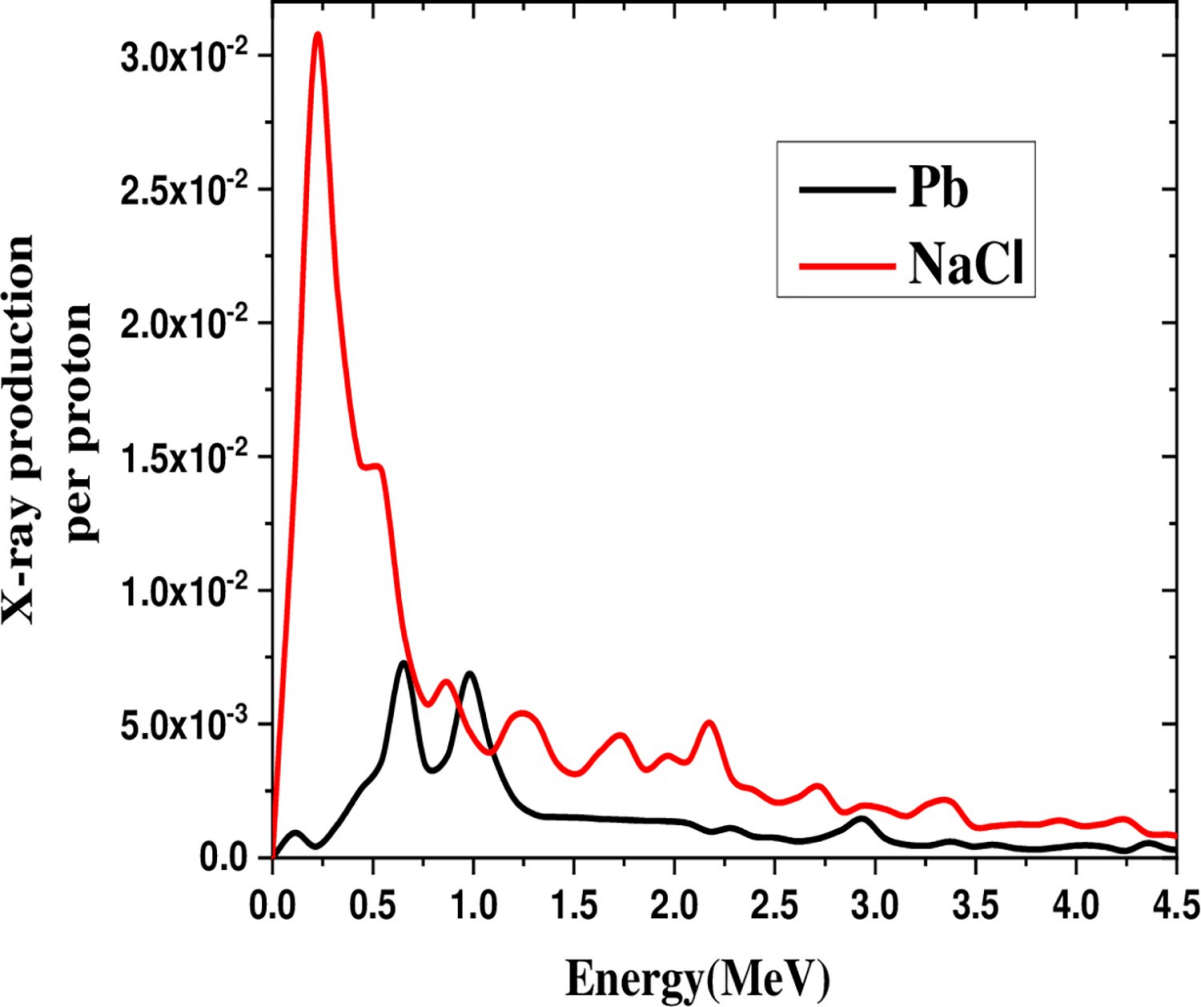
Comparison of X-ray production in the 0–5 MeV range by irradiating 100 MeV proton beam on salt and lead.

Fig 8 depicts the X-ray production in the energy range of 0–5 MeV by irradiating a 100 MeV proton beam on salt and lead. It revealed that the salt spectrum peak at 0.3 MeV was six times higher than in lead.

**Fig 9:**
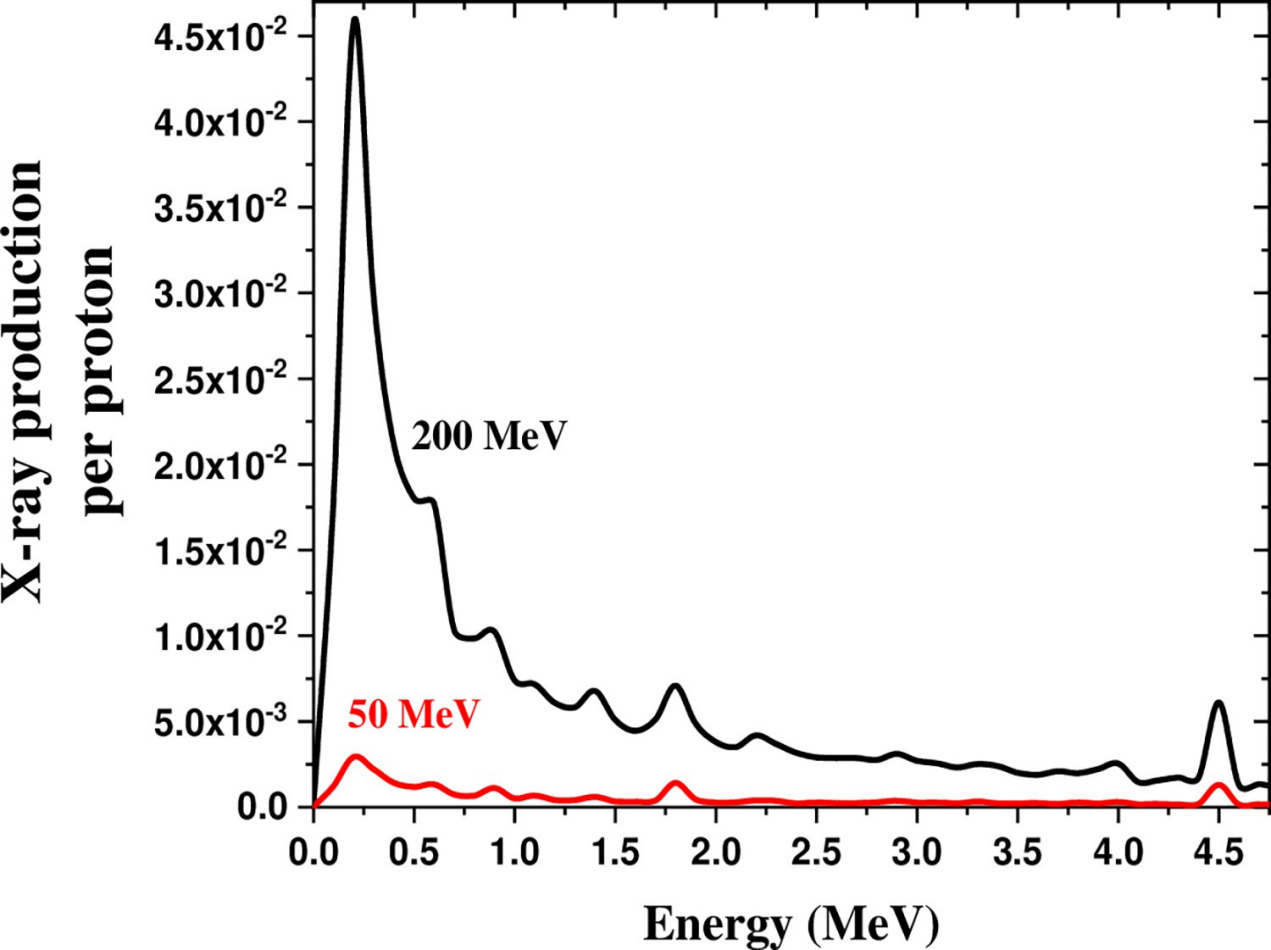
X-ray production in the energy range of 0–5 MeV by the 50 and 200 MeV proton beams on soil.

Fig 9 displays the X-ray production in the 0–5 MeV range by irradiating 50 MeV and 200 MeV proton beams on the soil. The increment of energy by four times spiked the spectrum peak at 0.30 MeV by nearly 18 times.

**Fig 10:**
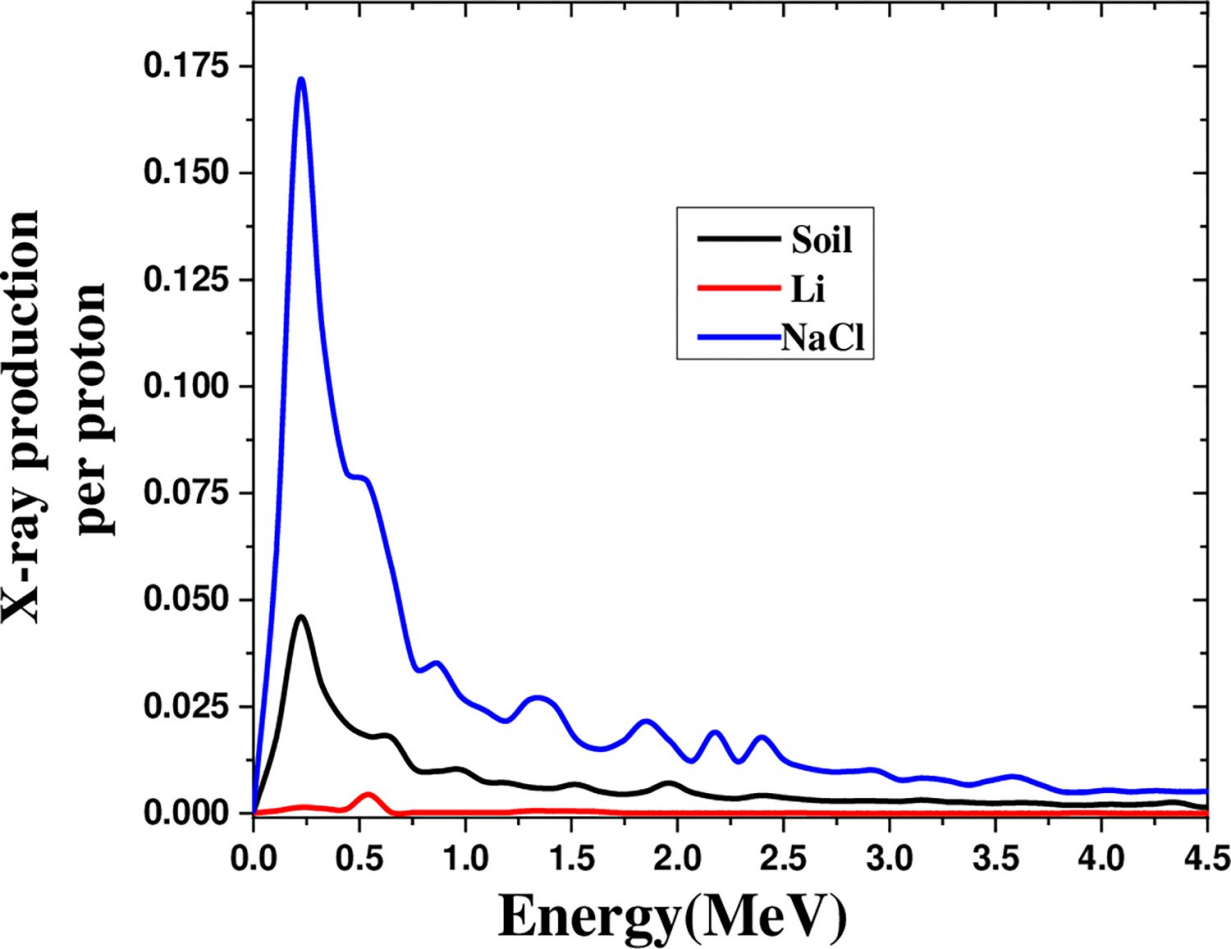
The X-ray production in the energy range of 0–5 MeV by the proton beam on soil, lithium, and salt in the energy threshold for spallation.

Fig 10 depicts the X-ray production in the range of 0–5 MeV by a proton beam on salt, lithium, and soil at the threshold energy for spallation (Table 1). The spectrum peak for salt was six times than that of lithium. Besides that, salt yielded maximum X-ray production gain due to spallation at the threshold energy (Table 2).

**Table 2:** X-ray production through the spallation process using different materials.

Material	Mass Number	Density (g/cm^3^)	Threshold energy of spallation (MeV) [22, 23]	Proton beam energy (MeV)	X-ray production Gain at the peak energy	peak energy (MeV)	Most probability interval energy of produced X-ray (MeV)
Lead	207	11.34 [57]	1630	100–1650	0.18	0.87	0–2.5
Nickel	58	8.90 [58]	506	600	0.084	0.33	0–2.5
Lithium	7	0.53 [59]	39	40	0.00134	0.50	0–0.6
Carbon	12	2.30 [60]	90	600	0.008	0.22	0–1.85
Salt	-	2.17 [61]	159–294	30–317	0.17	0.22	0–0.4
Soil	-	2.26 [62]	90–491	50–200	0.046	0.30	0–2.94

## Discussion

Table 2 lists the results obtained for X-ray production by the spallation process using light and heavy materials.

The heavy elements yield higher X-ray production than light elements as the proton energy is increased. Contrarily, the light elements have less X-ray production gain than heavy elements with decreased proton energy. In the proton energy range of 159–294 MeV, the X-ray production gain through spallation in salt was almost equal to that of lead with a proton energy of 1650 MeV. Based on the X -rays produced through proton spallation, it is recommended to use salt, soil, lead, and nickel. The results indicated that salt and lead with maximum X-ray production gain at 0.22 MeV (0–0.4 MeV range) and 0.87 MeV (0–2.5 MeV range) are good candidates for detecting elements via the PIXFE method.

## Conclusion

X-ray has many applications in science and technology. There are different ways to produce X-rays. One of the methods is to stop charged particles in other targets. When charged particles collide with atoms and nuclei, they are excited and emit X-rays and gamma-rays. To date, limited studies have investigated the effect of spallation on X-ray production. This study investigated the possibility of producing hard X-rays by activating the proton spallation process with different energies. During the spallation process, the target nuclei were evaporated by absorbing the energy of incident particles such as protons. In the process of nuclear evaporation, hard X-rays were also emitted in addition to producing nuclei with a mass number lower than the target mass number. For this purpose, the hard X-ray production was investigated due to proton beam irradiation on heavy, light, and mixed targets without considering the produced nuclei. Lead and nickel were the heavy, and lithium and carbon were the light nuclei used in this study. The combination of light nuclei that was used was NaCl. Furthermore, soil was chosen as the combination of heavy and light nuclei. The spallation process in heavy nuclei is possible when the energy of incident protons are higher than the proton energy of light nuclei, depending on the binding energy of nuclei. Consequently, the heavy lead and nickel nuclei have been irradiated by protons in the range of energy 100–1600 MeV, and 600 MeV, respectively, to produce a hard X-ray.

On the other hand, light carbon and lithium nuclei were irradiated with 600 and 40 MeV proton beams to produce hard X-rays. Also salt and soil were irradiated by a proton beam in the energy range of 30–317 MeV and 50–200 MeV, respectively, to produce hard X-rays. Based on the results, the hard X-ray spectrums were in the energy range of 0–8 MeV, where their peaks depended on the material and proton beam energy. The hard X-ray spectrum produced by the heavy elements with high-energy protons was similar to the hard X-ray spectrum produced by the light elements with low-energy protons. Hence, it was concluded that salt and lead yielded the maximum hard X-ray production gain due to the proton spallation. Salt was the best candidate for hard X-ray production due to the lower energy proton beam and higher hard X-ray production gain than the other materials. For 317 MeV proton irradiation on salt, the most probability interval energy of produced hard X-ray is 0–0.4 MeV. The hard X-ray production gain at peak energy (0.22 MeV) was 0.17. Therefore, 0–0.4 MeV energy for hard X-ray by proton irradiation on salt is a good candidate for detecting elements by the PIXFE method.

## Supporting information

S1 File (ZIP)
